# Study of Sonographic Indicators of Ovarian Reserves in Women With WHO-Defined Anovulatory Disorders

**DOI:** 10.7759/cureus.64741

**Published:** 2024-07-17

**Authors:** Punit Hans, Anjana Sinha

**Affiliations:** 1 Obstetrics and Gynaecology, Patna Medical College, Patna, IND; 2 Obstetrics and Gynaecology, Nalanda Medical College and Hospital, Patna, IND; 3 Obstetrics and Gynaecology, Patna Medical College and Hospital, Patna, IND

**Keywords:** ovarian reserve, antral follicle count, ovulatory dysfunction, who anovulatory classification, anovulatory infertility

## Abstract

Depending on the age of the wife or female partner, infertility is defined as the inability of a couple to achieve conception after 12 months if the age is ≤35 years and six months if the age is ≥35 years. About half of female infertility is attributed to ovulatory dysfunction, and the rest is either due to tubal and pelvic pathology or problems such as thyroid disease or anatomic abnormalities. Ovulatory disorders are one of the major factors for infertility problems in couples. Ultrasound monitoring of ovarian follicle growth plays a pivotal role in infertility treatment. The total number of small antral follicles in both ovaries is an important predictive parameter of the ovarian induction cycle, whereas ovarian volume is the parameter most easily assessed by ultrasound. The purpose of this study is to analyze the sonographic indicators of infertility (antral follicle count and ovarian volume) in four World Health Organization (WHO)-defined anovulatory groups and compare the same with that of healthy fertile women.

## Introduction

Over the past century, a global increase in economic and social development coinciding with a substantial decline in female fertility has been observed. The inability of a couple to achieve conception after 12 months of unprotected coitus is defined as infertility. When couples fail to achieve pregnancy after at least one year of unprotected sexual intercourse, then infertility is described as primary, whereas secondary infertility is described as when couples have been pregnant at least once but have not been able to achieve pregnancy again. About 8 to 15% of all women are estimated to experience primary or secondary infertility in their reproductive lives [1]. Over the past two decades, there have been significant changes in infertility practices. The introduction of in vitro fertilization (IVF) and other assisted reproductive technologies (ARTs) has raised the success rates of infertility treatment and also provided the opportunity to understand the basic reproductive process. ARTs include all the interventions involving direct retrieval of oocytes from the ovary. Ovulation failure is a major contributor (approximately 40%) of female infertility, whereas tubal and pelvic pathology contributes another 40%. Less than 10% comprises anatomic abnormalities or thyroid disease [2]. Of all the infertility problems in couples, approximately 20% are due to ovulatory disorders [2]. According to the WHO Classification of Anovulation (WHO Scientific group, Geneva 1976, Report 514), four anovulatory groups have been devised: (1) type I - hypogonadotropic hypogonadal anovulation (5-10%), which includes craniopharyngiomas, pituitary adenomas, arteriovenous malformations, or other central space-occupying lesions, Sheehan syndrome, and congenital hypothalamic failure (Kallmann syndrome); (2) type II - normogonadotropic normoestrogenic anovulation (75-80%), which includes polycystic ovarian syndrome (PCOS) (80%), adult-onset congenital adrenal hyperplasia, adrenal tumors, and androgen-producing ovarian tumors; (3) type III - hypergonadotropic hypoestrogenic anovulation (10-20%), which includes Turner syndrome, premature/autoimmune ovarian failure, and chemo/radiotherapy-induced ovarian damage; and (4) type IV - hyperprolactinemic anovulation. PCOS has been recognized as one of the most common endocrine/metabolic disorders in women. Its prevalence depends in part upon the diagnostic criteria used to define the disorder. Women with PCOS constitute the largest group of anovulatory women we encounter in our clinical practice (70 to 85% of cases). In a 2016 meta-analysis of 24 population studies performed in Europe, Australia, Asia, and the United States, the rate of PCOS (at the 95% confidence interval), according to the Rotterdam criteria, was 10% (8 to 13%, n = 15 trials) [3].

The number of ovarian follicles with a mean diameter ranging from 2 mm to 10 mm observed through transvaginal ultrasound during the early follicular phase of the menstrual cycle from cycle days 2 to 5 is recorded as the antral follicle count (AFC). The correlation of the AFC with the quantity of remaining ovarian follicles and the response of the ovaries to controlled ovarian stimulation have been demonstrated with good inter-cycle and inter-observer variability [4,5]. When there are only three to six total antral follicles, it is considered a low AFC, as evidenced by a meta-analysis study in which a low AFC had a mean of 5.2 (with 2.11 standard deviation) total AFC [4]. A low AFC is associated with a poor ovarian response to ovarian stimulation during IVF cycles; however, its predictability for conception failure is unreliable [4]. According to a study, the AFC was found to be the most important ovarian response and IVF outcome predictor in patients with normal basal serum follicle-stimulating hormone (FSH) levels [6]. The total number of small antral follicles in both ovaries is now considered the most important predictive parameter of cycle outcome and is also strongly related to reproductive age because it reflects the size of the remaining follicle pool.

One of the most easily assessed ovarian parameters is the ovarian volume. One study showed that the ovarian volume, assessed by transvaginal ultrasound, has predictive importance for ovarian response to ovulation induction [7]. In another study done later by Lass et al. [8], a strong association between the ovarian volume and ovarian reserve was shown, and it was recommended to measure the ovarian volume in all patients prior to IVF. The calculation of the ovarian volume requires ovarian measurements in three planes and the use of the ellipsoid formula: D1 x D2 x D3 x 0.52. The mean ovarian volume (MOV), the average volume calculated for both ovaries from the same individual, is the value used to access ovarian reserves. When screening for diminished ovarian reserves with imaging, the ovarian volume has limited value compared with the AFC for the detection of diminished ovarian reserves. However, a low ovarian volume (≤3 cm3) predicts a poor response to ovulation induction. Another important use of ovarian volume assessment is to differentiate between multifollicular and polycystic ovaries [9].

The aim of this study is to analyze the sonographic indicators (implying total AFC and ovarian volume) of ovarian reserves among the four WHO-defined anovulatory groups, comparing the same with that of healthy fertile women.

## Materials and methods

Source of data

This study was conducted as a prospective hospital-based study among patients coming to a gynecology OPD in Nalanda Medical College and Hospital, Patna, Bihar, India, with complaints of infertility from December 2014 to November 2016. The study was approved by the Institutional Review Board of Nalanda Medical College (no. OBG/1677, dated 2/11/2014), and informed consent was obtained from all participants. Among 211 infertile women (aged 19-45) from the attending investigation of subfertility enrolled for the study, after excluding other causes of infertility (e.g., tubal factor, uterine factor, pelvic factor, and male factor), the Anovulatory Infertility Group was formed. Healthy women (N = 25) belonging to the same age group (19-45 years) were enrolled from the patients being referred for problems unrelated to infertility or routine health check-ups, serving as the control group. The Anovulatory Infertility Group was further classified into four groups, according to the WHO classification of anovulatory disorders based on hormonal profile, clinical examination, and sonography findings. type I - hypogonadotropic hypogonadal anovulation, type II - normogonadotropic normoestrogenic anovulation, type III - hypergonadotropic hypoestrogenic anovulation, and type IV- hyperprolactinemic anovulation. The diagnosis of PCOS (included in the type II anovulatory disorder) was done on the basis of the presence of at least two of the criteria mentioned in the Rotterdam Classification, devised by the Rotterdam ESHRE/ASRM-sponsored PCOS Consensus Workshop Group in 2004: (1) ovulatory disturbances, mainly oligomenorrhoea or amenorrhoea; (2) clinical hyperandrogenism, defined by hirsutism (modified Ferriman-Gallwey score ≥8) or hyperandrogenism, defined biochemically by a serum testosterone level ≥0.7 ng/ml; and (3) ultrasound findings of >12 follicles in the 2 to 9 mm range in each ovary and/or ovarian volume >10 ml.

Recruitment

The exclusion criteria for the Anovulatory Infertility Study Group were as follows: (1) continued use of hormonal contraception or stopped two months prior to the study, (2) urine pregnancy test positive, (3) use of an oocyte donor, (4) any treatment for ovulation induction currently or earlier, and (5) critically ill patients.

The inclusion criteria for the Control Study Group were (1) regular menstrual cycles varying from 21 to 35 days and (2) at least one viable pregnancy carried to the term, and each of the pregnancies arose spontaneously within one year of the start of unprotected intercourse.

The exclusion criteria for the Control Study Group were (1) using hormonal contraception or stopped just two months before the start of the study, (2) the presence of ovarian abnormalities during ultrasonography, (3) any history of ovarian surgery, and (4) evidence of any endocrinal disease.

Study procedures 

Clinical screening included physical examination, menstrual and reproductive history, body mass index (BMI), surgery, and previous medication. Hirsutism was confirmed if the modified Ferriman-Gallwey score was 8 or higher [10]. BMI was calculated as weight (kg) divided by height squared (m^2^).

The endocrine screening included measurements on days 2-3 of the menstrual cycle of serum FSH, luteinizing hormone (LH), estradiol, testosterone, TSH (thyroid stimulating hormone), prolactin, and two-hour post-prandial blood sugar after intake of 75 gm glucose. From all patients after an overnight fast, peripheral blood flow was obtained between 8 a.m. and 11 a.m. In day 3, normal range values taken were FSH (3-20 mIU/mL), LH (2-7 mIU/mL), estradiol (25-75 pg/mL), TSH (3-5 mIU/mL), and prolactin (2-24 ng/mL).

Transvaginal sonography was done on days 2-3 of the menstrual cycle. All sonography measurements were performed using the 7.5 MHz transvaginal probe. All echo-free structures in the ovaries with a mean diameter (of two dimensions) of 2-5 mm and 6-10 mm in size were measured and counted separately in each ovary. Larger structures were considered cysts. The sum of counts in both the ovaries was the total AFC, which was obtained by transvaginal ultrasound at initial consultation in women with amenorrhoea, and between days 2 and 4 of the menstrual cycle for others. The MOV was calculated as the addition of left and right ovarian volume divided by two. Single ovarian volume was calculated using the formula based on a prolate ellipsoid: volume = π/6 × diameter 1 (maximum longitudinal) × diameter 2 (anteroposterior) × diameter 3 (transverse).

Regarding the statistical analysis, all the data were recorded and analyzed on a personal computer using IBM SPSS Statistics for Windows, Version 12 (Released 2004; IBM Corp., Armonk, New York, United States). Mean age, AFC, MOV, and BMI were tabulated and compared between the three groups and within the ovulatory group. A chi-squared test was used for non-parametric variables, and a p-value was calculated using chi-squared test-taking controls as a reference. Overall, p < 0.05 was considered significant for all statistical analyses. Correlations were determined using a Pearson-ranked correlation coefficient.

## Results

Among a total of 211 investigated cases of infertility, 55 (26.1%) cases were of anovulatory disorders, and five cases were lost in follow-up and hence excluded from the study. Among anovulatory disorders, the incidence of type I was 5.77% (3/52), type II was 71.11% (37/52), type III was 15.38% (8/52), and type IV was 7.69% (4/52). Moreover, type II comprised most polycystic ovarian disease at 94.5% (35/37), and only one case was of an adrenal tumor and one was of an androgen-producing ovarian tumor).

Table 1 shows the BMI distribution in the cases and controls. BMI was found to be on the higher side among cases.

**Table 1:** BMI distribution

BMI	Control (N = 25)	Anovulatory group (N = 52)
Underweight (<18.5 kg/m^2^)	0	3/52 (5.7%)
Normal (18.5–24.9 kg/m^2^)	20/25 (80%)	26/52 (50%)
Overweight (25–29.9 kg/m^2^)	5/25 (20%)	22/52 (42.3%)
Obese (≥30 kg/m^2^)	-	1/52 (1.9%)

Table 2 compares the correlation of BMI with the AFC and MOV between the control group and type II anovulatory disorder.

**Table 2:** Correlation of BMI with the antral follicle count and mean ovarian volume (Pearson coefficient) PCOS: Polycystic ovarian syndrome; AFC: antral follicle count; MOV: mean ovarian volume

Study Groups	AFC (2–5 mm)	AFC (6–10 mm)	MOV
Control	R = -0.4894	R = -0.3754	R = -0.542045
Type II anovulatory disorder (PCOS)	R = -0.048,	R = -0.08	R = 0.0712

In Table 3, the mean AFC and MOV were compared between different study groups. The mean AFC for follicles of small diameters (2-5 mm) was highest in type II (PCOD) anovulatory disorder (13.571), followed by type I anovulatory disorder (10) and type IV anovulatory disorder (5.25). It was lowest in type III anovulatory disorder (1.875). The mean AFC for follicles of diameter 6-10 mm was lowest in type I anovulatory disorder (0) and highest in the control group (4.08), whereas the MOV was highest in type II (PCOD) anovulatory disorder (12.02 mL) and lowest in type III anovulatory disorder (3.0375 mL). The p-value (calculated by using the Chi-squared test and taking controls as a reference) for each study group was highly significant for type II (PCOD) anovulatory disorder (p < 0.0038) and type III anovulatory disorder (p < 0.0058).

**Table 3:** Comparison of the mean antral follicle count and mean ovarian volume within study groups AFC: Antral follicle count; MOV: mean ovarian volume

Study Groups	AFC (2–5 mm)	AFC (6–10 mm)	MOV (ml)	p
Control	9.12	4.08	5.26	-
Type I anovulatory disorder	10	0	4.866	0.123
Type II (PCOD) anovulatory disorder	13.571	2.97	12.02	0.0038
Type III anovulatory disorder	1.875	0.25	3.0375	0.0058
Type IV anovulatory disorder	5.25	3	4.725	0.362

## Discussion

In our study, the incidence of anovulatory disorder (26.1%) in infertile couples was comparable to previous studies [11]. However, the incidence of undiagnosed infertility (12.8%) was slightly on the lower side, which might be explained by differences in the characteristics of the populations studied. Incidence of various types of anovulatory disorders (as defined by the WHO in 1976) in our study was type I (hypogonadotropic hypogonadal anovulation) at 5.77%, type II (normogonadotropic normoestrogenic anovulation) at 71.11%, type III (hypergonadotropic hypoestrogenic anovulation) at 15.38%, and type IV (hyperprolactinemic anovulation) at 7.69%. These findings were similar to those of Kousta et al. [12]. WHO type 2 anovulatory disorder almost exclusively applies to PCOS. A large specialist center study reported that 91% of women with WHO type 2 anovulation met the broader diagnostic criteria for PCOS [13], which was almost similar to our study results (94.5%). The slightly higher incidence in our study can be explained as PCOS is the most common endocrine disorder in women and a major cause of anovulation. Factors such as the diagnostic criteria and ethnic background of the population have a high influence on the prevalence of PCOS in the study population. Both PCO (polycystic ovary) and PCOS are more common in Indian women than in those from Northern Europe [14].

BMI distribution (Table 1) in our study showed that 42.3% of cases with ovulatory disorder were overweight, similar to the studies done by Majumdar et al. [15] and Ramanand et al. [16]. As most of the studies [17] on ovarian reserve and infertility have been done on Western populations, standard cut-off points set by these studies could not be applied to Indian populations. Thus, we compared the mean AFC (2-5 mm), AFC (6-10 mm), and MOV among cases and controls with the same population profile. Table 3 shows the mean AFC (2-5 mm) of 9.12, mean AFC (6-10 mm) of 4.08, and MOV at 5.26 mL for the controls in accordance with Agarwal et al. [18]. The mean AFC (2-5 mm) count was on the higher side (13.571), and so was the MOV (12.02 mL) in polycystic ovarian disease, with a highly significant p-value (0.0038), comparable with the findings by Bili et al. [19] and Battaglia et al. [20]. A high AFC (2-5 mm) was found to be more ardently associated with polycystic ovarian disease [21]. Type III anovulatory disorder showed the least value for mean AFC (2-5 mm) 1.875, mean AFC (6-10 mm) 0.25, and MOV (3.0375 mL), strongly correlating with poor ovarian reserves and premature ovarian failure [22]. The p-value was highly significant (0.0058). In our study, BMI showed a weak negative correlation for AFC in all the study groups, in accordance with the study by Malhotra et al. [23].

Both reference and experiment groups were from the same population, and hence they provided for a better comparison study. Low cost and better follow-up opportunities were strengths of the study. Factors serving as limitations in the study were the single study center and referral bias.

## Conclusions

By comparing differences in the mean AFC and MOV among study groups, we found that AFC (2-5 mm) and ovarian volume assessment by high-resolution transvaginal ultrasonography can be of significant value in diagnosing type II and type III anovulation disorder and also in deciding the choice of protocol for ovulation induction in various anovulatory infertility disorders.

Female fertility is influenced by physical, emotional, and social factors, including traditional and cultural values, which are vastly different in India from Western countries. There was a significant difference in Indian women’s mean AFC from Western recorded AFC, which can be attributed to different racial, geographic, and socioeconomic reasons. A large randomized study is needed for the Indian subcontinent to set standard cut-off values of the mean AFC and MOV for accessing ovarian reserves by ultrasonography. By using the standard cut-off values, we can better apply sonography to study correlations of age, BMI, FSH, and various other factors with ovarian reserves in the Indian population context. Considering the modern trend of postponement of pregnancy by women, along with a rising trend in obesity, there is a need to evaluate ovarian reserves to counsel couples seeking ARTs and decide on stimulation protocols.
